# Draft genome sequences for a *Staphylococcus aureus* and a *Staphylococcus haemolyticus* isolate from nasal swab samples from healthy females

**DOI:** 10.1128/mra.00518-24

**Published:** 2024-07-16

**Authors:** Sandra Jablonska, Hannah Brown, Helen Appleberry, Catherine Putonti, Alex Kula

**Affiliations:** 1 Department of Biology, Loyola University Chicago, Chicago, Illinois, USA; 2 Bioinformatics Program, Loyola University Chicago, Chicago, Illinois, USA; Department of Biological Sciences, Wellesley College, Wellesley, Massachusetts, USA

**Keywords:** *Staphylococcus aureus*, *Staphylococcus haemolyticus*, nasal microbiome

## Abstract

*Staphylococcus aureus* and *Staphylococcus haemolyticus* are common members of the human microbiota, but they are also opportunistic pathogens. To identify antibiotic resistance in healthy individuals, we present the genome sequences of *S. aureus* 139 N-1 and *S. haemolyticus* 173 N-3, both isolated from nasal swab samples from asymptomatic female participants.

## ANNOUNCEMENT

*Staphylococcus aureus* is a prominent member of the human microbiota, nasally colonizing about 30% of the human population asymptomatically (1, 2). *Staphylococcus haemolyticus* also colonizes the skin and nose asymptomatically, albeit at a lesser frequency (3). Antibiotic resistance and multidrug resistance is a mounting concern for both species (3 – 5). In this study, we present two draft genome assemblies*—S. aureus* 139 N-1 and *S. haemolyticus* 173 N-3. Both strains were isolated from nasal swabs of healthy female participants.

Participants in our institutional review board (IRB)-approved study (IRB no. 3603) self-identified as female, “healthy,” and had not taken antibiotics within the last 6 months. Participants were students at Loyola University Chicago (Chicago, IL) and the two isolates described here are from two different, unrelated individuals. Each participant was instructed to rub the cotton swab (BD BBL CultureSwab) on the lower inside of the nostril for 30 seconds, after which the swab was inserted into the provided storage solution (Amies media). Within 1 hour of sampling, swabs were swirled and squeezed in the storage media and then spread on mannitol salt (MS) agar plates, which is a selective media for staphylococci, and incubated for 24 hours at 35°C with 5% CO_2_. Morphologically distinct colonies were selected, placed in MS broth, and incubated under the same conditions. This process was repeated to purify the isolates. DNA was extracted using the DNeasy blood and tissue kit (Qiagen), following the manufacturer’s protocol for Gram-positive bacteria. Library construction was performed by SeqCoast using the Illumina DNA Prep tagmentation kit and unique dual indexes and then sequenced on the Illumina NextSeq2000 platform using a 300-cycle flow cell kit (2 × 150 bp reads). Read demultiplexing, read trimming, and run analytics were performed using DRAGEN v3.10.12 prior to delivery from SeqCoast. Assembly was performed using the BV-BRC v3.35.5 web tool with the “auto” option (6). First, raw reads were trimmed using trim_galore v0.6.5dev (https://github.com/FelixKrueger/TrimGalore) and assembled using Unicycler (v0.4.8; 7). Then, assemblies were polished using Pilon (v1.23; 8); three rounds of polishing were performed for the *S. aureus* 139 N-1 assembly and two rounds for the *S. haemolyticus* 173 N-3 assembly. BV-BRC also was used to calculate the genomic coverage. Taxonomy was confirmed using the Type Strain Genome Server (TYGS) (9), and digital DNA–DNA hybridization (dDDH) for both strains exceeded the species threshold. The genome assemblies were annotated by the NCBI Prokaryotic Genome Annotation Pipeline (PGAP) v6.7 (10), and completeness and contamination were computed by CheckM v1.2.2 (11) upon submission to the NCBI. Antibiotic resistance genes were identified using ResFinder v4.5.0 (12, 13), specifying the *“Staphylococcus aureus”* species parameter. Default parameters were used for all software tools, except for those specified here.

Table 1 lists the assembly statistics for the two genomes. Given the concern of antibiotic resistance among these two species, we screened the genome assemblies for antibiotic resistance genes. *S. aureus* 139 N-1 encodes for *blaZ*, suggesting resistance to penicillin. *S. haemolyticus* encodes for *msrA* and *mphC*, both of which are associated with resistance to macrolides. Further testing is needed to confirm these predictions.

**TABLE 1:** Genome assembly statistics for *S. aureus* 139 N-1 and *S. haemolyticus* 173 N-3

Strain	139 N-1	173 N-3
Species designation	*S. aureus*	*S. haemolyticus*
TYGS dDDH (d4%) [type strain]	77.8 [GCF_002902425.1]	91.3 [GCF_002901805.1]
Assembly length (bp)	2,832,477	2,443,656
G + C (%)	32.74	32.70
No. of contigs	69	70
Contigs N50 (bp)	150,667	93330
Coverage (x)	142.52	137.14
Completeness (%)	97.74	99.94
Contamination (%)	0.71	0.02
No. of reads	3,100,328	2,723,114
SRA accession no.	SRR28706147	SRR28706148
Assembly accession no.	JBCGEB000000000	JBCGEC000000000

## Data Availability

Table 1 lists the accession numbers for the raw reads and assemblies.
